# Review of Surgical Cases Treated in the Buffalo General Hospital from March to July, 1888

**Published:** 1888-09

**Authors:** Herman Mynter

**Affiliations:** Professor of Surgery in Niagara University; Surgeon to the Hospital of the Sisters of Charity, and to the Emergency Hospital. (Late Surgeon Buffalo General Hospital.)


### Buffalo Medical and Surgical Journal

Vol. XXVIII. SEPTEMBER, 1888. No. 2

### Original Communications.

### *REVIEW OF SURGICAL CASES TREATED IN THE BUFFALO GENERAL HOSPITAL FROM MARCH TO JULY, 1888.**

By HERMAN MYNTER, M. D.,

Professor of Surgery in Niagara University; Surgeon to the Hospital of the Sisters of Charity, and to the Emergency Hospital. (Late Surgeon Buffalo General Hospital.)

Gentlemen—I have thought it might interest you more to listen to a short report of the work done in the surgical ward of the General Hospital during my term just finished, than a more lengthy paper on arthrectomy, for which I was booked. I will reserve that for the future.

The number of patients who entered the hospital during my four months' service, on account of surgical diseases, not counting those whom I found as a legacy from my predecessor, was 153; 128 males and 25 females. Of these, 117 recovered, 2 improved, 3 were not benefited, 11 died, and 20 were left over in the ward.

The mortality was, therefore, a little above seven per cent., or not counting those who died inside of twenty-four hours from severe injuries (four), a little more than four per cent. Of the seven left, one died of pyæmic abscesses following perinephritis; one of cirrhosis of the kidneys (entered hospital on account of contusion of the back); one died one-half hour after amputation of the femur on account of gangrene, the result of a compound

* Read before the Buffalo Medical and Surgical Association, August 7, 1888.

and comminuted fracture of the leg, but he was almost pulseless before the operation, with cold extremities; one died in thirty-six hours from heart failure after amputation of the femur on account of diffuse gangrene (senile); one of peritonitis thirty-six hours after herniotomy for strangulated femoral hernia of four days' duration; one of Bright's disease, and lastly, one six hours after laparotomy on account of ileus of seven days' duration. In no case, therefore, to use the same words which occasioned such a commotion in this Association a few years ago when the report of the Sisters' Hospital was published, was death the direct result of operative measures.

The number of operations performed was sixty-nine, of which fifty-five recovered, one improved, six died, and seven were left over in the hospital. I wish here to add that our antiseptic measures consisted exclusively in corrosive sublimate dressings, prepared in the hospital itself; that not a single wound has been infected, in spite of the large number of cases of erysipelas treated in the hospital—perhaps the cause of the great mortality in the maternity ward—and that healing by first intention has been the invariable rule, even after such operations as arthrectomy and amputation of femur.

I desire here to review some of the more interesting cases and operations.

Of abscesses and kindred diseases there were thirteen cases, some of which were of formidable nature, and one deserves special mention. The patient was a man twenty-six years of age, who three days after a spree was attacked with severe pain in left side, followed with constant soreness, high temperature, and a diffuse, erysipelatous-looking swelling of the whole lateral region of the chest. On entering, an incision was made four inches long between the twelfth rib and *crista ilei*. The subcutaneous tissue was infiltrated with pus, but no distinct abscess found. The wound was plugged with iodoform gauze under an antiseptic bandage. For some days he felt better, but the temperature then increased to 104½° in the evening and 101° in the morning, with pyæmic symptoms, delirium and great prostration. The diffuse swelling continued, becoming a little more doughy and crepitant. Under ether-narcosis therefore an incision was made reaching from the *crista ilei* to the axilla, about one foot long. All the subcutaneous tissues were found gangrenous, and several pounds of dead tissue were removed with scissors from almost a square foot of surface. Even the connective tissue between the subscapular muscle and the *serratus anticus* muscles was removed more or less.

The operation was altogether the most formidable of its kind, which either a large number of physicians who were present or I myself ever had seen; the patient left the table all but dead, and was sustained for several hours by hypodermic injections of brandy and camphor before the pulse could be felt at the wrist. The wound was dressed with iodoform gauze and an antiseptic sublimate dressing, which was changed once a week. His recovery was retarded by a number of large pyæmic abscesses in the right arm, forearm, left infraclavicular region, and left calf; but the enormous wound is now healed, and the patient healthy and ready to leave the hospital.

Of amputations, nine were made: One of the humerus on account of crushed arm; two Chopart's for crushed feet; one of a finger for tuberculous tendonitis; one of the leg for tuberculous inflammation of ankle-joint and four of the femur, of which two died. They were both amputated on account of gangrene of the leg; in one case the gangrene was occasioned by a compound and comminute fracture of the leg, and the patient survived the operation, which was done as a last measure, but one-half hour. The other case was that of a woman thirty-eight years of age, who entered with gangrene of the whole foot and lower third of leg, of unknown cause. It had come on suddenly, had all the appearances of *gangrena senilis*, although she was not prematurely old, and had lasted for eight days. The patient was much prostrated, but improved immediately after the amputation. The high temperature fell to normal, the quick pulse (140) became slower, but she died suddenly thirty-six hours after the amputation with symptoms of heart failure.

No cause was found at the post mortem for the gangrene, and the arteries particularly were healthy. One case of amputation was of special interest. The patient was a man forty-four years of age, who had been more or less crippled for thirty years on account of injury to his right femur. Abscesses had frequently formed and necrotic bones discharged. Six months ago spontaneous fracture occurred. He declined any other operation than amputation. Several fistulas led to the fracture, which had taken place in the lower third of the femur. The operation was done with two lateral flaps. A number of sequestra were found, the whole upper part of the *os femoris* was in a state of osteomyelitis, and the contents of the bony cavity were removed up to the trochanter major with a sharp spoon, so that the outer thin shell only was left. The cavity was packed with iodoform gauze left in position under the antiseptic bandage for two weeks. Yet the wound healed throughout by first intention, the medullary canal closing in a week after the removal of the iodoform gauze.

Complete arthrectomy, on account of tubercular arthritis, was done in two cases—one of the elbow and one of the knee. Both cases are of interest. The first case was that of a young girl twenty years of age, who had for many months had a diseased elbow-joint with pain, swelling, muscular atrophy, crepitus and lateral motion. Complete arthrectomy of the elbow was made through two lateral incisions. Three weeks later, on account of necrosis of the lower epiphysis of the humerus, resection of both epiphyses was performed, they being made to protrude through the old incisions. Thereafter healthy granulation and rapid healing took place. The result is excellent, with almost perfect flexion and extension, and without lateral motion. The patient has not yet been discharged.

The second case was that of a little boy, ten years of age, who underwent, in one operation, arthrectomy of the knee-joint, resection of the epiphyses and a large sequestrotomy of the corresponding femur. He struck the right knee upon a stone six months before, and was thereafter confined to his bed for a few days. Shortly after he was attacked by what his physicians called erysipelatous blood-poisoning, was delirious for two weeks, and an abscess formed in left forearm. Shortly after, an incision was made on inner side of left femur, and a large amount of pus and broken-down tissue evacuated. He probably had an acute osteomyelitis at that time.

The wound never healed, and continued to discharge yellow thick pus. On admission, the knee-joint was found stiff, bent at an angle of 45°; a sinus was found over inner side of femur, leading to dead bone; some lateral motion in the knee-joint, which had the usual appearance of a tuberculous joint; no crepitation. March 10, 1888—operation. The joint was opened with a downward convex incision through the *ligamentum patellæ inferioris.* Patella, femur and tibia were found in a state of caries, the capsule was much thickened and studded with tubercles, and was entirely removed with the patella. A large cavity was found in the lower third of femur that was chiseled open, and a sequestrum two and a-half inches long by three-quarters of an inch broad removed. A slice of femur, three-quarters of an inch thick, and one of tibia, one-half inch thick, were removed with the saw, in order to stretch the limb. The wound was sutured with catgut—no drainage tubes were used—but the wound left partially open at the lowest lateral points. It was covered with protective, then a thick corrosive sublimate bandage; lastly, of plaster of Paris bandage. Thereafter the Esmarck bandage was removed and the joints filled with antiseptic blood after Schede's method.

April 3d, first change of bandage. Wound healed by first intention, except over internal condyle, where there is found a small fistula, leading into dead bone, which was loose and was removed.

Three more dressings were made, and the patient was in the middle of June almost well, and commenced to walk well by aid of crutches. There is still a small fistula left, but without sequestrum.

One operation was made on account of a contracted cicatrix in the palm of the hand, by which the thumb was strongly adduced, and the hand useless.

The cicatrix was dissected out and everything contracted cut through without regard to the anatomy, and the wound healed by Schede's blod-clot method. The result was perfect.

Six cases were operated for *fissura* and *fistula ani.* In all the sphincter ani muscle was violently stretched (Recamier's method), and the fistulas then cut through. After-treatment consisted in sitz-baths for one-half hour morning and night, and thereafter iodoform meché. All recovered promptly.

Seven cases of tumors were treated, of which six were operated, and recovered. Two cases had lymphoma of neck and inguen; one a large fibro-sarcoma of the forearm, taking its origin from the periosteum of the ulna; one had a large ganglion in the popliteal region as large as a goose-egg; it was connected with the tendon of the semitendinosus muscle, and the wound healed by first intention; one case had a large bunion over the metatarso-phalangeal joint, which was removed and healed by transplantation.

One patient, a little girl, had a relapse of a retrobulbar sarcoma, for which evisceration of all the contents of the eye-cavity, even the periosteum, was performed. No relapse has so far occurred, and the cavity is rapidly filling up by granulations from the bone itself, and will, I suppose, be perfectly obliterated. It might also have been treated by Schede's blood-clot method.

One case had its peculiar interests. The patient was a man fifty-six years of age, with a good family history. He had for nine months had pain in the right thigh, and loss of muscular power, and was treated twice in the medical ward for chronic rheumatism. During his stay in the hospital a spontaneous fracture of the right femur occurred, for which he was removed to the surgical ward. There was found a deep, hard swelling of the middle third of the femur, evidently connected with the periosteum, and being an osteo-sarcoma. Exarticulation of the hip-joint was proposed, but declined. He staid four weeks in the hospital, during which time the sarcoma increased rapidly. Thereafter he was removed to the almshouse.

Of operations on the venous system, two were performed. One was an extirpation of a troublesome varicocele by free incision and thereafter ligating the convolute of veins above and below with catgut, and then cutting them out. The wound healed by first intention; but an orchitis developed shortly after the operation, and delayed the recovery. Possibly the *vas deferens* was pinched during the operation.

The other case was a total phlebectomy, or extirpation of an enormously dilated *vena saphena magna* through an incision reaching from the knee to the ankle. The wound healed by first intention, and all his troublesome symptoms have disappeared.

One case of herniotomy for strangulated femoral hernia of four days' duration terminated fatally in thirty-six hours from peritonitis.

One case of laparotomy for internal obstruction was interesting. The patient, a man forty-nine years of age, had had inflammation of the bowels twenty years ago, and had since suffered, more or less, from dyspepsia and constipation. Four days before entering the hospital he was complaining of meteorismus and increasing abdominal pain. On entering he had constant pain, tenesmus and tenderness in abdomen, especially in the epigastric region, accompanied with tympanitis and fœcal vomiting, and he had had no movement of the bowels for a week. On the medical ward he was for two days treated with hot poultices, enemata with turpentine, ice and milk, but as fœcal vomiting set in he was removed to Gates's Cottage on March 6th. After preliminary washing out of the stomach, laparotomy was performed through a median incision. Without difficulty the occlusion was found in the lower part of the ileum, near cœcum. It was produced by a band of old cicatricial tissue, which was doubly ligated and cut through. The outer coat of the intestines ruptured transversely in several places, and was sutured with catgut. The intestines were badly congested and very brittle, and the patient survived but six hours. If the operation had been performed earlier, the probability is that he would have recovered, so much more, as he was operated under the same conditions and in the same place in which the ovariotomies are done.

Two staphyloraphies for defects of the palate were made. One was a complete congenital cleft palate in a man thirty-five years of age, and the result was perfect. His speech is greatly improved, although yet nasal. The other, also successful, was on account of a large defect in the hard palate from syphilis. Two cases of resection for coxitis deserve mention.

One was a girl eleven years of age, who was healthy till three months ago. Without known injury, she woke up one morning with pain in left hip and knee and unable to walk. She had been confined to her bed since, lost greatly in weight, and had starting pains at night. At entrance she was very much emaciated. Left foot was adducted, flexed and rotated inward. Trochanter major was found two inches above Nelaton's line. Flexion was moderately free, extension and outward rotation impossible. The head of the femur was plainly felt on the ileum. Resection of the hip was performed above trochanter minor. The head of the femur was found deprived of its cartilage, and the bony canals found open and granulating. There was no trace of the round ligament. The acetabulum was greatly enlarged upward and backward, and in a carious condition. It was scraped out and the whole capsule extirpated. By rectal examination there was found thickening under the *obturatur internus* muscle, but no abscess. The patient is still in the hospital, but was progressing fast towards recovery when I left. The interest in this case is particularly the early occurrence of dislocation of the femur.

The other case was a man twenty-four years of age, who had been more or less sick and unable to walk for two years after being struck by a railroad switch. He entered with a large cold, abscess on the front of the thigh, and the usual symptoms of coxitis. On March 7th the abscess was opened, and the abscess membrane was found studded with tubercles. The abscess could be followed into the joint, which was resected above trochanter minor. *Caput femoris* and acetabulum were found rough and carious; the capsule was tuberculous and was removed. He has since had, now and then, returns of tuberculous material in the wound, which has shown little inclination to heal, and the prognosis was, when I left the hospital, still uncertain.

A case of necrosis of the symphysis pubis, in which the whole symphysis was removed, was interesting. The patient was a man twenty-three years of age, who, three years ago, fell from a car, striking on a switch-handle one-half inch in front of anus, producing a wound five inches deep into the pelvic tissue and fracturing the right ischium, necessitating removal of parts of the ischium. He spent four months in the Sisters' Hospital before the wound was healed. Four months ago he discovered a swelling over the pubis which gave him great pain, was accompanied with continual fever and inability to walk. An abscess formed at last, which was opened but showed no inclination to heal. The fistula was at last laid open, and a little opening found, which led inwards to the symphysis. The opening was enlarged by cutting over the attachment of the pyramidales muscles, and the symphysis pubis was found necrotic, and about one inch on both sides of the symphysis was removed from the horizontal part of *os pubis.* After the operation a finger, introduced into the wound, could be quite severely pinched by pressure on the *cristæ iliorum.* He has since steadily improved and commences to walk quite well, although the wound is not yet healed..

Two operations of external urethrotomy and one of internal urethrotomy were successfully performed.

Twenty-two cases of various kinds of fractures entered the hospital, of which three died (one from fracture of skull, one from fracture of the vertebræ with complete paralysis, and one from compound and comminuted fracture of the leg, followed by gangrene; he survived amputation of the femur but half an hour). The rest recovered. For fracture of the femur I have for some time in every case used Volkman's sliding splint with fifteen to twenty-pound weight, and no coaptation-splints, and there has been a minimum of shortening (less than one-half inch). For fractures of the leg I employ plaster of Paris, applied second day over a snugly-fitting stocking. The result has uniformly been good.

For compound and comminuted fractures I always open freely the wound, remove loose splinters and all contused and suspicious looking tissues, and try to heal the wound by Schede's blood-clot method. I have only seen one unfavorable result, the patient dying after amputation of the femur from gangrene of the leg. The question was one of primary amputation or conservative treatment, and we erred, if error there was, on the side of conservatism.

Six cases of erysipelas were treated and recovered, but no wound was infected from them.

For lacerated wounds of hands and feet I have used permanent water-baths with excellent results. Two patients with crushed feet, who entered to have amputation performed, recovered with useful limbs, and another, who had got his hand caught between two rollers and received all kinds of injuries (broken fingers, open joints, torn tendons and lacerated wounds), recovered with an almost perfect hand by the use of permanent water-bath with corros. sublimate (1—10,000).

The condition of the hospital is excellent, the nursing perfect, and, altogether, an operation can be performed there with a minimum of risk. Following are the tables, showing diagnosis and results of the operations :

### DIAGNOSIS.

| DIAGNOSIS. | Male. | Female. | Total. | Recovered. | Improved. | Not Improved. | Died. | In Hospital. |
|---|---|---|---|---|---|---|---|---|
| Abscess—Axillary | 1 | . . | 1 | . . | . . | . . | . . | 1 |
| " facial | . . | 1 | 1 | 1 | . . | . . | . . | . . |
| " gluteal | 1 | . . | 1 | 1 | . . | . . | . . | . . |
| " leg | 1 | . . | 1 | 1 | . . | . . | . . | . . |
| " submaxil | 1 | . . | 1 | 1 | . . | . . | . . | . . |
| " perineal | 1 | . . | 1 | 1 | . . | . . | . . | . . |
| " regiones lat. pectoris | 1 | . . | 1 | 1 | . . | . . | . . | . . |

### DIAGNOSIS—Continued.

| DIAGNOSIS. | Male. | Female. | Total. | Recovered. | Improved. | Not Improved. | Died. | In Hospital. |
|---|---|---|---|---|---|---|---|---|
| Abscess—Perinephritic | 1 | | 1 | | | | *1 | |
| Bubo | 2 | | 2 | 2 | | | | |
| Burn—Arm | 1 | | 1 | 1 | | | | |
| " leg | 1 | | 1 | 1 | | | | |
| Caries—Tarsi | 2 | 1 | 3 | 3 | | | | |
| " tibia | | 1 | 1 | 1 | | | | |
| Cellulitis—Diffuse, of hand and arm | 1 | | 1 | 1 | | | | |
| " of chest | 1 | | 1 | | | | | 1 |
| Cleft palate | 2 | | 2 | 2 | | | | |
| Concussion brain | 3 | | 3 | 2 | | | †1 | |
| Contusion—Back | 2 | | 2 | 1 | | | ‡1 | |
| " breast | 1 | | 1 | 1 | | | | |
| " knee | 2 | | 2 | 1 | | | | 1 |
| " abdomen | 1 | | 1 | 1 | | | | |
| Coxitis | 1 | 1 | 2 | | | | | 2 |
| Cicatricial contraction thumb | 1 | | 1 | 1 | | | | |
| Bunion | 1 | | 1 | 1 | | | | |
| Crush—Of arm | 1 | | 1 | 1 | | | | |
| " of foot | 2 | | 2 | 2 | | | | |
| " of thigh and leg | 1 | | 1 | | | | §1 | |
| Dislocation—Of elbow | 1 | | 1 | 1 | | | | |
| " clavicle, acromial end | 1 | | 1 | | | | | 1 |
| Empyema | 1 | | 1 | 1 | | | | |
| Epididymitis | 3 | | 3 | 3 | | | | |
| Erysipelas—Faciei | 5 | 1 | 6 | 6 | | | | |
| Fissura—Ani | 3 | 1 | 4 | 4 | | | | |
| Fistula—Ani | 3 | | 3 | 3 | | | | |
| " urethra | 1 | | 1 | | | | | 1 |
| Fractura—Compound tibia and fibula | 1 | | 1 | | | | ‖1 | |
| " skull | 1 | 2 | 3 | 2 | | | °1 | |
| " femur | 2 | | 2 | 1 | | 1 | | |
| " humerus, surg. neck | | 1 | 1 | 1 | | | | |
| " radius and ulno | 1 | | 1 | 1 | | | | |
| " ribs | 4 | | 4 | 3 | | | | 1 |
| " tibia | 1 | | 1 | 1 | | | | |
| " tibia and fibula | 2 | 3 | 5 | 4 | | | | 1 |
| " nose | 1 | | 1 | 1 | | | | |
| " vertebræ dorsi | 3 | | 3 | | | | **1 | 2 |
| Gangrene—Leg | | 1 | 1 | | | | ††1 | |
| " senile | 1 | | 1 | | | | | 1 |
| Ganglion—Reg. poplitœa | 1 | | 1 | 1 | | | | |
| Harelip | 1 | | 1 | 1 | | | | |
| Hemorrhage—From varicose vein | 1 | | 1 | 1 | | | | |
| Hernia—Cruralis incarcerata | | 1 | 1 | | | | ‡‡1 | |
| Ileus | 1 | | 1 | | | | §§1 | |
| Mastitis—Puerperal | | 1 | 1 | 1 | | | | |
| Necrosis—Ilei and sacri | 1 | | 1 | | | | ‖‖1 | |
| " femoris | 2 | | 2 | 2 | | | | |
| " tarsi | 1 | | 1 | 1 | | | | |
| " scapulæ | 1 | | 1 | 1 | | | | |
| " symphysis pubis | 1 | | 1 | | | | | 1 |

### DIAGNOSIS—CONTINUED.

| DIAGNOSIS. | Male. | Female. | Total. | Recovered. | Improved. | Not Improved. | Died. | In Hospital. |
|---|---|---|---|---|---|---|---|---|
| Paralysis—Infantilis | . . | 1 | 1 | . . | . . | 1 | . . | . . |
| Periostitis—Acute | . . | 1 | 1 | 1 | . . | . . | . . | . . |
| " calcanei | 2 | . . | 2 | . . | . . | . . | . . | 2 |
| Pelveo-peritonitis—Localis | 1 | . . | 1 | 1 | . . | . . | . . | . . |
| Pedes valgi | 1 | . . | 1 | . . | 1 | . . | . . | . . |
| Pott's disease—Cervical | 1 | 1 | 2 | 2 | . . | . . | . . | . . |
| Prostata, Hypertrophy of | 1 | . . | 1 | . . | 1 | . . | . . | . . |
| Rupture—Of abdominal muscles | 1 | . . | 1 | 1 | | . . | . . | . . |
| Sprain—Of ankle | 1 | . . | 1 | 1 | . . | . . | . . | . . |
| Strictura—Urethræ | 7 | . . | 7 | 7 | . . | . . | . . | . . |
| Synovitis, Chronic—Of knee | 1 | 1 | 2 | 2 | . . | . . | . . | . . |
| " " of elbow | . . | 1 | 1 | . . | . . | . . | . . | 1 |
| " suppurative of ankle | 1 | . . | 1 | . . | . . | . . | . . | 1 |
| Syphilis—Secondary | 4 | 1 | 5 | 5 | . . | . . | . . | . . |
| " tertiary | 2 | . . | 2 | . . | . . | . . | . . | 2 |
| Tendinitis tubere | 1 | . . | 1 | 1 | . . | . . | . . | . . |
| Tumors—Lymphoma of neck | 1 | . . | 1 | 1 | . . | . . | . . | . . |
| " " of inguen | 1 | . . | 1 | 1 | . . | . . | . . | . . |
| Sarcoma retro-bulbosa | . . | 1 | 1 | 1 | . . | . . | . . | . . |
| Fibrosarcoma antibrachii | 1 | . . | 1 | 1 | . . | . . | . . | . . |
| Osteosarcoma femoris | 1 | . . | 1 | . . | . . | 1 | . . | . . |
| Epithelioma labii sup. | . . | 1 | 1 | 1 | . . | . . | . . | . . |
| Ulcer—Indolent | 1 | 1 | 2 | 2 | . . | . . | . . | . . |
| " leg, chronic | 2 | . . | 2 | 2 | . . | . . | . . | . . |
| " syphilitic | 5 | . . | 5 | 5 | . . | . . | . . | . . |
| " varicose | 1 | . . | 1 | 1 | . . | . . | . . | . . |
| " tropho-neurotic | . . | 1 | 1 | . . | . . | . . | . | 1 |
| Varicose veins | 1 | . . | 1 | 1 | . . | . . | . . | . . |
| Wound—Lacrated, of arm | 2 | . . | 2 | 2 | . . | . . | . . | . . |
| " " of hand | 1 | . . | 1 | 1 | . . | . . | . . | . . |
| " reg. parietalis | 1 | . . | 1 | 1 | . . | . . | . . | . . |
| " " occipit | 3 | . . | 3 | 3 | . . | . . | . . | . . |
| " " leg | 2 | . . | 2 | 2 | . . | . . | . . | . . |
| " of knee-joint | 1 | . . | 1 | 1 | . . | . . | . . | . . |
| " foot | 1 | . . | 1 | 1 | . . | . . | . . | . . |
| Varicocele | 1 | . . | 1 | 1 | . . | . . | . . | . . |
| Total | 128 | 25 | 153 | 117 | 2 | 3 | 11 | 20 |

* Pyæmic abscesses.
† Apoplexia, in twelve hours.
‡ Cirrhosis kidney.
§ In one-half hour after entering hospital.
‖ Gangrene, one-half hour after amputation of thigh.
° In twelve hours, of apoplexia.
** In twenty-four hours, with general paralysis.
†† Heart failure, thirty-six hours after amputation of thigh.
‡‡ Peritonitis, thirty-six hours.
§§ Six hours after laparotomy.
‖‖ Morbus Brightii.

### OPERATIONS.

| OPERATION. | Male. | Female. | Total. | Recovered. | Improved. | Not Improved. | Died. | In Hospital yet. |
|---|---|---|---|---|---|---|---|---|
| Abscess—Opened and curetted | 10 | 3 | 13 | 13 | . . | . . | . . | . . |
| Amputations—Arm | 1 | . . | 1 | 1 | . . | . . | . . | . . |
| " Chopart | 2 | . . | 2 | 2 | . . | . . | . . | . . |
| " finger | 1 | . . | 1 | 1 | . . | . . | . . | . . |
| " leg | 1 | . . | 1 | 1 | . . | . . | . . | . . |
| " thigh | 3 | 1 | 4 | 2 | . . | . . | *2 | . . |
| Arthrectomy of elbow | . . | 1 | 1 | . . | . . | . . | . . | 1 |
| " " knee | 1 | . . | 1 | 1 | . . | . . | . . | . . |
| Caries—Scapula, curetted | 1 | . . | 1 | 1 | . . | . . | . . | . . |
| " tarsi | 1 | 1 | 2 | 2 | . . | . . | . . | . . |
| " tibia | . . | 1 | 1 | . . | . . | . . | . . | 1 |
| Castration | 1 | . . | 1 | 1 | . . | . . | . . | . . |
| Contracted cicatrix of hand, extirpated | 1 | . . | 1 | 1 | . . | . . | . . | . . |
| Empyema operation | 1 | . . | 1 | 1 | . . | . . | . . | . . |
| Fissura, ani dilatation | 1 | 1 | 2 | 2 | . . | . . | . . | . . |
| Extirpation—Lymphoma neck | 2 | . . | 2 | 2 | . . | . . | . . | . . |
| " fibro-sarcoma antibrachii | 1 | . . | 1 | 1 | . . | . . | . . | . . |
| " ganglion, reg. poplitœa | 1 | . . | 1 | 1 | . . | . . | . . | . . |
| " bunion hallucis | 1 | . . | 1 | 1 | . . | . . | . . | . . |
| " fistula ani | 4 | . . | 4 | 4 | . . | . . | . . | . . |
| " varicocele | 1 | . . | 1 | 1 | . . | . . | . . | . . |
| Evisceration eye cavity | . . | 1 | 1 | 1 | . . | . . | . . | . . |
| Explorative incision scalp | 1 | . . | 1 | . . | . . | . . | †1 | . . |
| Herniotomy | . . | 1 | 1 | . . | . . | . . | ‡1 | . . |
| Hydrocele, injection carbolic acid | 2 | . . | 2 | 2 | . . | . . | . . | . . |
| Laparotomy—For ileus | 1 | . . | 1 | . . | . . | . . | §1 | . . |
| Incision and drainage of joints | 1 | . . | 1 | . . | . . | . . | . . | 1 |
| Plastic operation—Cleft palate | 2 | . . | 2 | 2 | . . | . . | . . | . . |
| " " harelip | 1 | . . | 1 | 1 | . . | . . | . . | . . |
| Puncture knee-joint and sublimate injection | 2 | . . | 2 | 2 | . . | . . | . . | . . |
| Phlebectomy—Vena saphena magna | 1 | . . | 1 | 1 | . . | . . | . . | . . |
| Resection—Elbow | . . | 1 | 1 | . . | . . | . . | . . | 1 |
| " hip | 1 | 1 | 2 | . . | . . | . . | . . | 2 |
| Sequestrotomy—Femur | 1 | . . | 1 | 1 | . . | . . | . . | . . |
| " symphysis pubis | 1 | . . | 1 | . . | . . | . . | . . | 1 |
| Skin-grafting, after Thiersch | 2 | 1 | 3 | 2 | 1 | . . | . . | . . |
| Trephining skull | 1 | 1 | 2 | 1 | . . | . . | ‖1 | . . |
| Urethrotomia—Externa | 2 | . . | 2 | 2 | . . | . . | . . | . . |
| " interna | 1 | . . | 1 | 1 | . . | . . | . . | . . |
| Total | 55 | 14 | 69 | 55 | 1 | 0 | 6 | 7 |

* (1) Amputated in dying condition from gangrene ; lived one-half hour. (2) Died thirty-six hours after amputation for gangrene, of heart failure.

† Apoplexia.

‡ Peritonitis, in thirty-six hours.

§ Six hours, of shock.

‖ Inside twenty-four hours.

